# Decreased Paneth cell α-defensins promote fibrosis in a choline-deficient L-amino acid-defined high-fat diet-induced mouse model of nonalcoholic steatohepatitis via disrupting intestinal microbiota

**DOI:** 10.1038/s41598-023-30997-y

**Published:** 2023-03-09

**Authors:** Shunta Nakamura, Kiminori Nakamura, Yuki Yokoi, Yu Shimizu, Shuya Ohira, Mizu Hagiwara, Zihao Song, Li Gan, Tomoyasu Aizawa, Daigo Hashimoto, Takanori Teshima, Andre J. Ouellette, Tokiyoshi Ayabe

**Affiliations:** 1 Graduate School of Life Science, Hokkaido University, Sapporo, Japan; 2 Hokkaido Laboratory, Corporate Research & Development Division, Nitto Denko Corporation, Sapporo, Japan; 3 Department of Cell Biological Science, Faculty of Advanced Life Science, Hokkaido University, Kita-21, Nishi-11, Kita-ku, Sapporo, Hokkaido 001-0021 Japan; 4 Department of Advanced Transdisciplinary Science, Faculty of Advanced Life Science, Hokkaido University, Sapporo, Japan; 5 Department of Hematology, Faculty of Medicine, Hokkaido University, Sapporo, Japan; 6 Department of Pathology and Laboratory Medicine, Keck School of Medicine, University of Southern California, Los Angeles, CA USA

**Keywords:** Immunology, Gastroenterology

## Abstract

Nonalcoholic steatohepatitis (NASH) is a chronic liver disease characterized by fibrosis that develops from fatty liver. Disruption of intestinal microbiota homeostasis, dysbiosis, is associated with fibrosis development in NASH. An antimicrobial peptide α-defensin secreted by Paneth cells in the small intestine is known to regulate composition of the intestinal microbiota. However, involvement of α-defensin in NASH remains unknown. Here, we show that in diet-induced NASH model mice, decrease of fecal α-defensin along with dysbiosis occurs before NASH onset. When α-defensin levels in the intestinal lumen are restored by intravenous administration of R-Spondin1 to induce Paneth cell regeneration or by oral administration of α-defensins, liver fibrosis is ameliorated with dissolving dysbiosis. Furthermore, R-Spondin1 and α-defensin improved liver pathologies together with different features in the intestinal microbiota. These results indicate that decreased α-defensin secretion induces liver fibrosis through dysbiosis, further suggesting Paneth cell α-defensin as a potential therapeutic target for NASH.

## Introduction

Nonalcoholic steatohepatitis (NASH) is a progressive chronic liver disease characterized by fat accumulation in the liver, hepatocyte damage, inflammation, and subsequent fibrosis, which progresses to cirrhosis and liver cancer^1^. Suppressing liver fibrosis is a major challenge in the treatment of NASH, because fibrosis increases the risk of liver-related mortality^2^. The fibrosis progression rate among nonalcoholic fatty liver disease (NAFLD) patients is about 40% and varies among individuals^3^. Thus, a multiple hit hypothesis has been considered in which NASH development proceeds through the complex crosstalk of varied factors not limited to the liver and that the process of NASH onset is diverse^4^. The progression of NASH pathology is associated with overnutrition^5^, yet the specific factors that lead to progression from nonalcoholic fatty liver to NASH have not been clarified.

The liver plays a central role in metabolism and directly communicates with the intestine to form the “gut-liver axis”^6^. The human intestine harbors forty trillion bacteria affecting a variety of host functions including energy acquisition and consumption, in which the intestinal microbiota form a symbiotic relationship with the host^7^. On the other hand, dysbiosis, disruption of intestinal microbiota homeostasis, is associated with many metabolic dysfunctions such as fatty liver and obesity^8,9^. Moreover, the composition and diversity of the intestinal microbiota are influenced by diets, and metabolites such as short-chain fatty acids (SCFA) regulate liver fatty acid syntheses of fatty acids and bile acids in the liver^10^. In addition, dysbiosis induces hepatic inflammation in NAFLD patients, in which alterations in bacterial metabolites and an influx of pathogen-associated molecular patterns promote chronic inflammation in the liver^11^.

The intestinal epithelial cells that separate the intestinal lumen from the underlying tissue are constantly exposed to food and microbes, absorbing nutrients needed for host survival and sending them to the liver while preventing invasion by pathogens^12^. Paneth cells, an epithelial lineage located at the base of small intestinal crypts, secrete granules rich in α-defensins, human defensin (HD) 5 in humans and cryptdins (Crps) in mice, into the intestinal lumen, and contribute to innate enteric immunity mainly by the potent microbicidal activities of α-defensins against pathogens^13–17^. Furthermore, α-defensins regulate composition of the intestinal microbiota by the selective bactericidal activities, killing pathogenic bacteria while being symbiotic with commensal bacteria^18^. The absence of activated Crps in matrix metalloproteinase 7-deficient mice increases the prevalence of Firmicutes and decreases Bacteroidetes in the small intestine, while the production of HD5 in *DEFA5* transgenic mice increases Bacteroidetes and decreases Firmicutes, indicating that α-defensins affect the intestinal microbiota composition^19^. Previous reports showed that Paneth cell α-defensins play a role in regulating the intestinal microbiota^20^ and that impaired Paneth cell granule secretion is associated with diseases such as Crohn’s disease, graft-versus-host disease (GVHD), and depression^21–24^.

In this study, we hypothesized that abnormalities in Paneth cells and their α-defensins induce dysbiosis, resulting in damages of the liver, and lead to liver fibrosis. Here we show, in choline-deficient, L-amino acid-defined, high-fat diet (CDAHFD)-induced NASH model mice, the quantity of α-defensins secreted from Paneth cells decreased significantly before the onset of NASH, resulting in dysbiosis with decreased microbiota diversity followed by fibrosis leading to NASH. Furthermore, restoring luminal levels of α-defensins by inducing Paneth cell regeneration by treatment with R-Spondin1 (R-Spo1)^25–27^ or by oral administration of a mouse α-defensin, Crp4, both suppress dysbiosis and ameliorate liver fibrosis. Our results show that dysbiosis associated with decreased Paneth cell α-defensin secretion contributes to disease onset and progression in the CDAHFD model of NASH, further providing insights into Paneth cell α-defensins in “gut-liver axis” in NASH fibrosis.

## Results

### Paneth cell α-defensin secretion decreases before onset of NASH in the CDAHFD group

**Figure 1:**
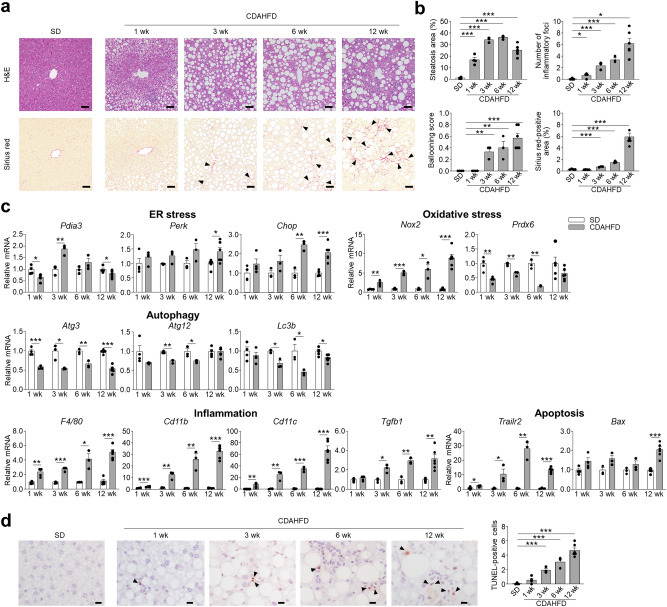
CDAHFD group develop liver injury and fibrosis associated with dysregulated expression of ER stress, oxidative stress, and autophagy related genes. (**a**) Representative images of hematoxylin and eosin (H&E)- and Sirius red-stained liver sections. Arrowheads indicate Sirius red-positive area. Scale bars: 50 μm. (**b**) Quantification of steatosis area, lobular inflammation, hepatocellular ballooning, and Sirius red-positive area. The degree of lobular inflammation was evaluated by counting the number of inflammatory foci. (**c**) Hepatic mRNA expression of ER stress, oxidative stress, autophagy, inflammation, and apoptosis related genes was analyzed by real-time PCR. (**d**) Representative images of TUNEL staining of liver sections (left). Arrowheads indicate TUNEL-positive cells. Scale bars: 10 μm. Quantification of TUNEL-positive cells (right). Data are shown as mean ± SEM for *n* = 3–6 per group. *P < 0.05, **P < 0.01 and ***P < 0.001, by unpaired two-tailed Student’s *t* test.

To investigate whether Paneth cell α-defensins are involved in the onset of NASH, we examined mice fed CDAHFD. First, histological analyses of the liver were conducted on mice fed standard diet (SD) or CDAHFD, fat accumulation and lobular inflammation were observed in the CDAHFD group at 1 week (wk), also both the area of steatosis and the number of inflammatory foci increased with wks. Hepatocellular ballooning increased in the CDAHFD group at 3 wk and continued to increase until 12 wk (Fig. 1a,b). Sirius red staining was performed to evaluate liver fibrosis, showing that Sirius red-positive fibrotic areas first appeared at 3 wk and continued to increase until 12 wk in the CDAHFD group (Fig. 1a,b). The number of inflammatory foci showed a strong positive correlation with fibrosis (Supplementary Fig. 1a). We further examined specific marker gene expressions of ER stress, oxidative stress, and impaired autophagy, known to be involved in NASH pathology^4^. Among ER stress markers, *Pdia3* mRNA expression decreased at 1 wk and increased at 3 wk, *Chop* increased at 6 and 12 wk, and *Perk* increased at 12 wk in the CDAHFD group compared to the SD group (Fig. 1c). Expression of *Nox2* mRNA, a key source of redox radicals, increased, and antioxidant enzyme *Prdx6* mRNA decreased after 1 wk in the CDAHFD group. Among autophagy activating protein genes, *Atg3* mRNA expression declined after 1 wk followed by decreasing expression of both *Atg12* and *Lc3b* mRNAs from 3 wk in the CDAHFD group. In addition, *F4/80* and *Cd11b*, markers of resident and invasive macrophages, respectively, and dendritic cell marker *Cd11c* mRNA levels continued to increase after 1 wk. Fibrosis related growth factor *Tgfb1* increased after 3 wk in the CDAHFD group. mRNA levels for *Trailr2* increased from 1 wk, and *Bax* increased at 12 wk (Fig. 1c). The number of terminal deoxynucleotidyl transferase–mediated dUTP nick end labeling (TUNEL)-positive liver cells in the CDAHFD group increased at 3 wk and continued to increase with progression of fibrosis up to 12 wk (Fig. 1d). The number of TUNEL-positive cells showed strong positive correlation with both the number of inflammatory foci and the area of fibrosis (Supplementary Fig. 1b). These results indicated that inflammation and apoptosis in the liver contributed to the onset and progression of fibrosis in the NASH model and that ER stress, oxidative stress, and impairment of autophagy are involved in these pathways.

**Figure 2:**
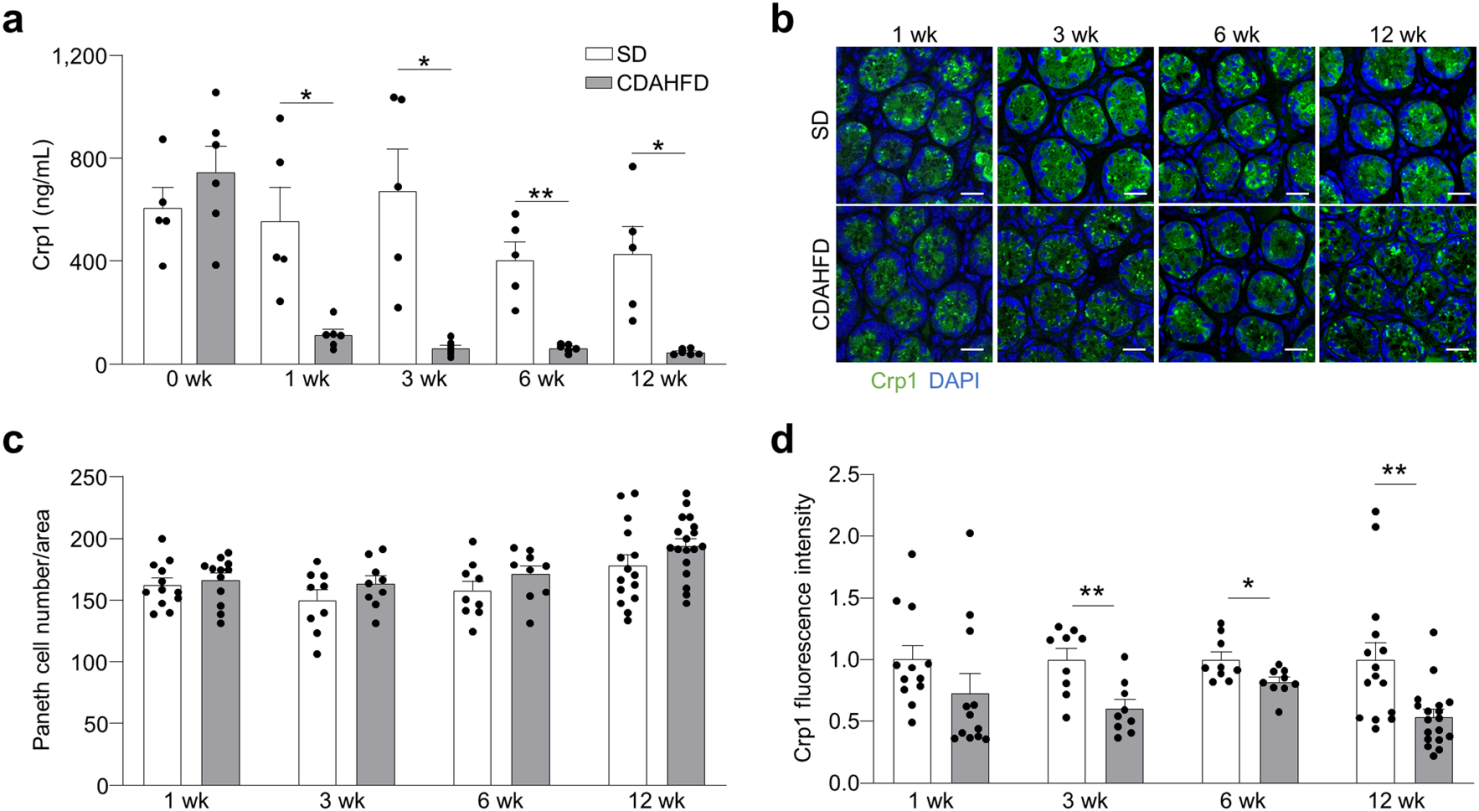
Secretion and protein expression of Paneth cell α-defensin decrease in the CDAHFD group. (**a**) Quantification of fecal levels of Crp1. Data are shown as mean ± SEM for n = 5–6 mice/group. (**b**) Representative images of immunofluorescence staining of Crp1 (green) in small intestine from SD and CDAHFD group. DAPI (blue) stains the nucleus. Scale bars: 20 μm. (**c**) Quantification of the number of Paneth cells and (**d**) Crp1 fluorescence intensity on Paneth cells shown in (**b**). Data are shown as mean ± SEM for* n* = 9–18 fields/group and quantified based on 3 fields per mouse. Each group contains 3–6 mice. *P < 0.05, **P < 0.01 and ***P < 0.001, by unpaired two-tailed Student’s *t* test.

Fecal levels of Crp1 were measured to evaluate the quantity of α-defensins secreted by Paneth cells. Crp1 is the most abundant α-defensins, in the mouse small intestine^28^. Fecal Crp1 levels in the CDAHFD group significantly decreased compared to the SD group before the onset of liver fibrosis, and continued for 12 wk (Fig. 2a). Furthermore, fecal Crp1 levels showed negative correlation with steatosis, inflammation, apoptosis, and fibrosis (Supplementary Fig. 2). To investigate the cause of decreased Crp1 secretion, we analyzed *Crp1* mRNA expression, Paneth cell number, and whole-mount fluorescence immunostaining of Crp1 on the ileal tissue samples. No differences were observed in both *Crp1* mRNA expression and Paneth cell number between the SD and CDAHFD groups (Fig. 2b,c, and Supplementary Fig. 3a,b). In contrast, fluorescent intensities of Crp1 in the CDAHFD group were diminished compared to SD after 3 wk (Fig. 2b,d).

### Paneth cell granule secretion is impaired in the CDAHFD group

**Figure 3:**
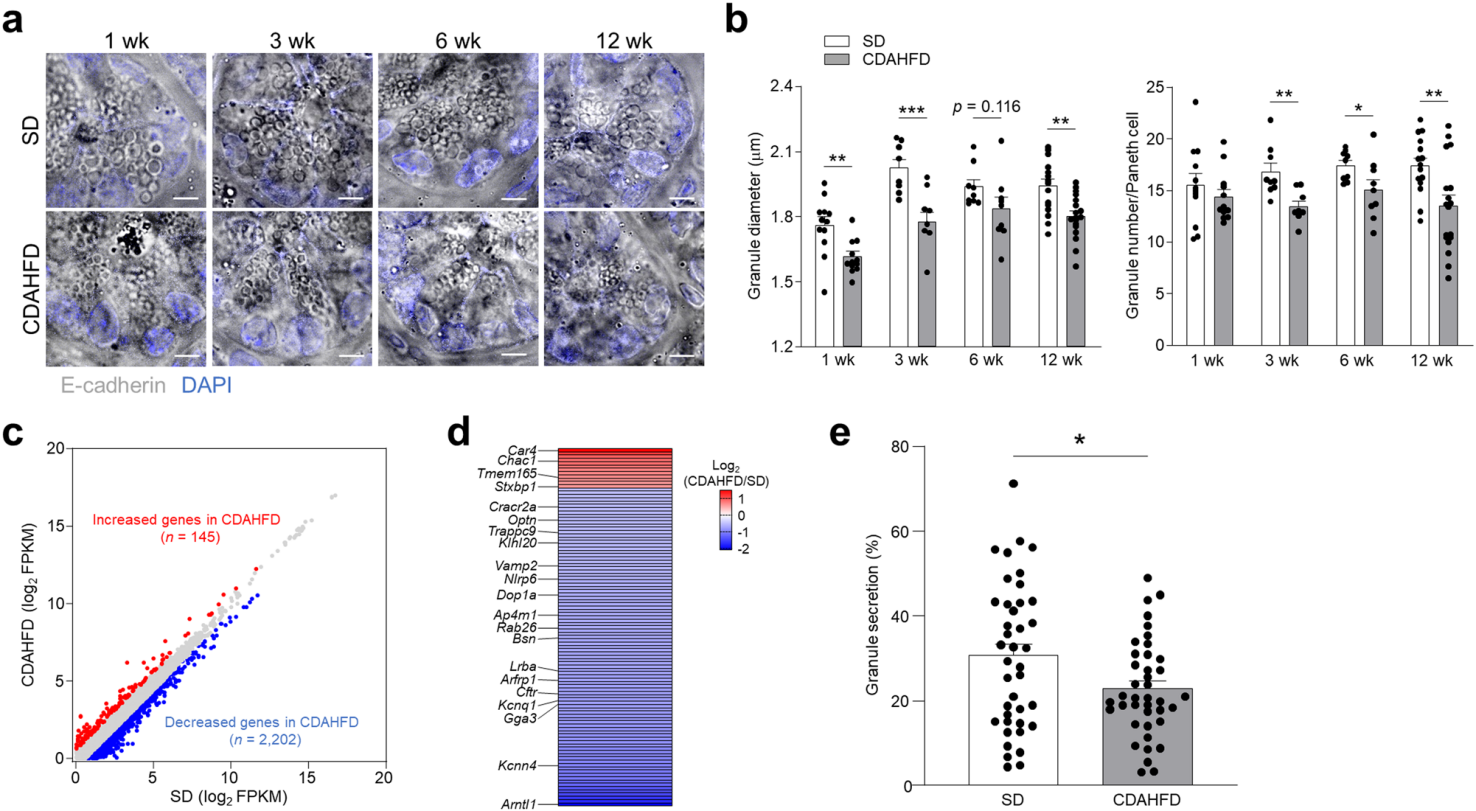
Paneth cell granule secretion is impaired in the CDAHFD group. (**a**) Representative images of whole mount small intestine obtained with confocal microscopy. Scale bar: 5 μm. (**b**) Granule diameter and granule number of each Paneth cell per field. Data are shown as mean ± SEM for *n* = 9–18 fields/group and quantified based on at least 3 fields per mouse. Each group contains 3–6 mice. (**c**,**d**) Paneth cells isolated from small intestine of mice fed with SD and CDAHFD for 3 wk were analyzed by RNA-seq. Paneth cells are pooled from 6 mice/group and data show the average of two independent experiments. (**c**) Scatterplot of global gene expression profiles of SD and CDAHFD group derived from RNA-seq analysis. (**d**) Heatmap showing log_2_ fold change of differentially expressed genes (1.5-fold increase or decrease) associated with Paneth cell functions in the CDAHFD group compared with SD group. (**e**) Enteroids derived from the small intestine of SD and CDAHFD groups were stimulated by 1 μM CCh for 10 min. Percent granule secretion was calculated as percent area granule secretion. Data are shown as mean ± SEM for *n* = 40 crypts/group. Each group contains 4 mice. *P < 0.05, **P < 0.01 and ***P < 0.001, by unpaired two-tailed Student’s *t* test.

Because α-defensins are abundant in Paneth cell granules normally, we examined the morphology of Paneth cell granules to possibly explain the decreased levels of α-defensins in Paneth cells of the CDAHFD group. The diameter of Paneth cell granules decreased after 1 wk of the CDAHFD group, and the number of Paneth cell granules also decreased after 3 wk (Fig. 3a,b). Transmission electron microscopy (TEM) images showed the abnormal morphologies of Paneth cell granules in the CDAHFD group (Supplementary Fig. 4). To further determine the molecular basis for the functional alterations in Paneth cells, RNA sequencing (RNA-seq) analysis was performed on isolated Paneth cells from both groups. One hundred and forty-five genes were upregulated in the CDAHFD group compared to the SD group, while 2,202 genes were downregulated (Fig. 3c). These differentially regulated genes were compared with gene products by referring to GO terms: Secretion (GO: 0046903) and trans-Golgi network (GO: 0005802), known to have key roles in the granule secretory pathway. Twelve upregulated genes in CDAHFD Paneth cells include ion transporter *Car4*, oxidative stress gene *Chac1*, glycosylation-related *Tmem165*, and secretion-related *Stxbp1*. Ninety-seven down regulated genes include ion transporters *Cracr2a*, *Kcnq1*, *Cftr*, and *Kcnn4*, vesicle transporters *Optn*, *Trappc9*, *Vamp2*, *Dop1a*, *Rab26*, *Lrba*, *Arfrp1*, and *Gga3*, autophagy-related *Klhl20*, *Nlrp6*, *Ap4m1*, and *Bsn*, and oxidative stress-related protein *Arntl1* (Fig. 3d and Supplementary Table 1). These results indicated that expression of genes related to the granule secretory pathway is dysregulated in Paneth cells of the CDAHFD group.

Abnormalities of ER stress, autophagy, and oxidative stress related genes are known to result in morphological changes in Paneth cell granules^21,29^, suggesting impairment of granule secretory function induced by CDAHFD. In addition, Paneth cells of the CDAHFD group showed dysregulated expression of genes essential for granule secretion including vesicle transporters and ion channel transporters. Thus, to evaluate Paneth cell granule secretion of the CDAHFD group directly, granule secretory responses of Paneth cells were visualized in enteroids, a three-dimensional culture system of small intestinal epithelial cells^17^. When carbachol (CCh), which induces Paneth cell granule secretion, was added to enteroid cultures derived from small intestinal crypts of the CDAHFD group, the quantity of granules secreted by Paneth cells was significantly lower than that of the SD group enteroids (Fig. 3e). These results indicated that Paneth cell dysfunction impairs granule secretion, resulting in diminished release of α-defensins into the intestinal lumen.

### Dysbiosis occurs along with decreased Crp1 secretion in the CDAHFD group

**Figure 4:**
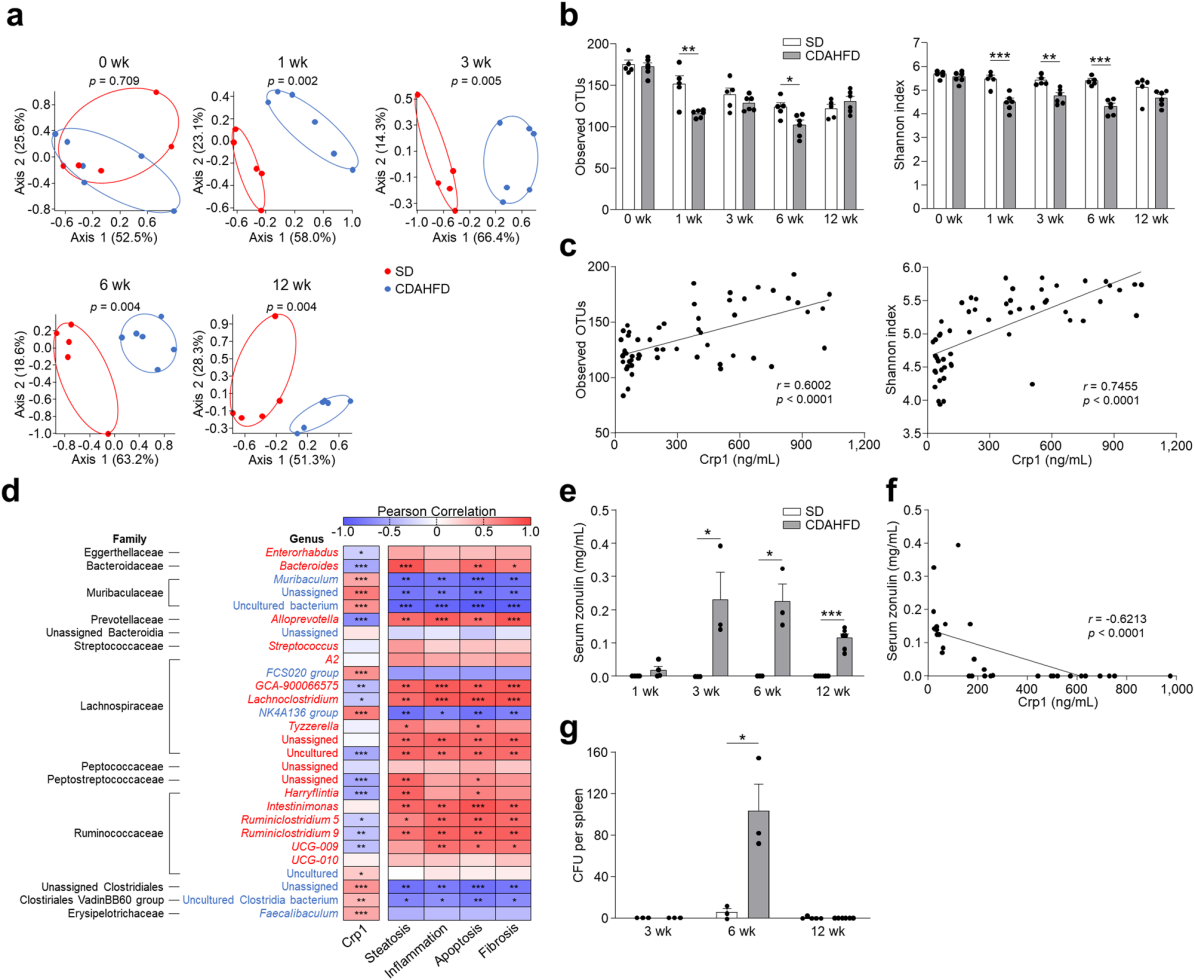
CDAHFD group shows dysbiosis correlated with both the quantity of α-defensin in feces and NASH pathology. (**a**) PCoA plot of intestinal microbiota based on weighted UniFrac distance of SD and CDAHFD groups. Significance was computed with PERMANOVA. (**b**) Observed OTUs and Shannon index in SD and CDAHFD groups. (**c**) Correlation analysis between fecal Crp1 levels and α-diversity indexes. (**d**) 28 genera that were significantly increased or decreased in the CDAHFD group compared with the SD group. Red indicates genera significantly increased in the CDAHFD group, and blue indicates genera significantly decreased in the CDAHFD group. Heatmap showing Pearson correlation coefficients between relative abundance of significantly changed genera in the CDAHFD group and fecal Crp1 levels from Fig. 2a or NASH pathology from Fig. 1b. Correlation analysis between Crp1 levels and relative abundance of individual genera was conducted using the data of 1, 3, 6, and 12 wk. Correlation analysis between NASH pathology and relative abundance of individual genera was conducted using the data of 12 wk. (**e**) Serum zonulin levels in SD and CDAHFD group. (**f**) Correlation analysis between fecal Crp1 and serum zonulin levels. (**g**) Quantification of bacteria in spleen by CFUs cultured from spleen of SD and CDAHFD group at 3, 6, and 12 w under anaerobic condition. Data are shown as mean ± SEM for *n* = 5–6 mice/group in (**a**,**b**,**c**,**d**). Data are shown as mean ± SEM for *n* = 3–6 mice/group in (**e**,**f**,**g**). *P < 0.05, **P < 0.01 and ***P < 0.001, by unpaired two-tailed Student’s *t* test (**b**,**e**,**g**) and Pearson correlation test (**c**,**d**,**f**).

To discern the effect of decreased Crp1 secretion on the intestinal microbiota, we conducted 16S rRNA gene sequencing on fecal DNA. Principal coordinate analysis (PCoA) showed that the bacterial community of the CDAHFD group formed a different cluster compared to the SD group at 1 wk and continued through 12 wk (Fig. 4a). α-Diversity analysis showed that observed operational taxonomic units (OTUs) and Shannon index were significantly decreased at 1 wk, and positive correlation was observed between fecal Crp1 levels and the α-diversity indexes, indicating that dysbiosis occurs before the onset of NASH in the CDAHFD group (Fig. 4b,c). At the family level, the relative abundances of Eggerthellaceae, Bacteroidaceae, Prevotellaceae, Streptococcaceae, Peptostreptococcaceae, and Ruminococcaceae were significantly increased in the CDAHFD group. In contrast, Muribaculaceae, unassigned family of unassigned Bacteroidia class, unassigned Clostridiales, Clostridiales VadinBB60 group, and Erysipelotrichaceae were significantly decreased relative to the SD group (Supplementary Fig. 5a). At the genus level, a total of 28 genera showed significantly different occupancy rates between the SD and CDAHFD group during disease progression, and among them, twenty genera showed a positive or negative correlation with fecal Crp1 (Fig. 4d). To determine whether the dysbiosis relates to disease progression, correlation analyses were conducted between the relative abundance of the intestinal microbiota and NASH pathology. The relative abundance of 10 genera which increased in the CDAHFD group, including *Bacteroides, Alloprevotella, GCA-900066575, Lachnoclostridium,* uncultured Lachnospiraceae*,* unassigned Peptostreptococcaceae*, Harryflintia, Ruminiclostridium 5, Ruminiclostridium 9,* and *UCG-009* correlated positively with NASH pathology including steatosis, inflammation, apoptosis, and fibrosis. In contrast, the 6 genera that decreased in abundance, *Muribaculum,* unassigned Muribacululaceae*,* Muribaculaceae uncultured bacterium*, NK4A136 group,* unassigned genus of the unassigned Clostridiales family*,* and uncultured Clostridiales VadinBB60 group negatively correlated with NASH pathology (Fig. 4d and Supplementary Fig. 5b). Furthermore, fecal Crp1 levels showed negative correlation with NASH pathology (Supplementary Fig. 2). These results suggested that the decrease of Crp1 secretion is associated with NASH development and progression via dysbiosis.

Because increased intestinal permeability associated with dysbiosis allows for translocation of enteric microbiota or bacterial components to the liver, contributing to NASH development, we next measured serum zonulin, a marker of intestinal permeability^30^. At 1 wk, the SD and CDAHFD group had similar serum zonulin levels. However, at 3 wk and continuing for 12 wk, serum zonulin levels in the CDAHFD group were increased significantly compared to SD mouse sera (Fig. 4e). To further assess whether the intestinal permeability increased, we analyzed mRNA expression of tight junction protein, and determined that the mRNA expression of claudin-1 decreased significantly in the ileum of the CDAHFD group at 3 and 6 wk (Supplementary Fig. 6a,b). Furthermore, serum zonulin showed negative correlation with fecal Crp1 levels, showing that dysbiosis induced by decreased Crp1 relates to increased intestinal permeability (Fig. 4f). In testing for bacterial translocation, bacterial colonies were detected in spleens at 6 wk in the CDAHFD group (Fig. 4g). Thus, these findings suggested that the dysbiosis associated with decreased Crp1 secretion resulted sequentially in intestinal hyperpermeability as well as bacterial translocation.

### R-Spo1 administration restores luminal α-defensin secretion, suppressing dysbiosis and reducing NASH progression

**Figure 5:**
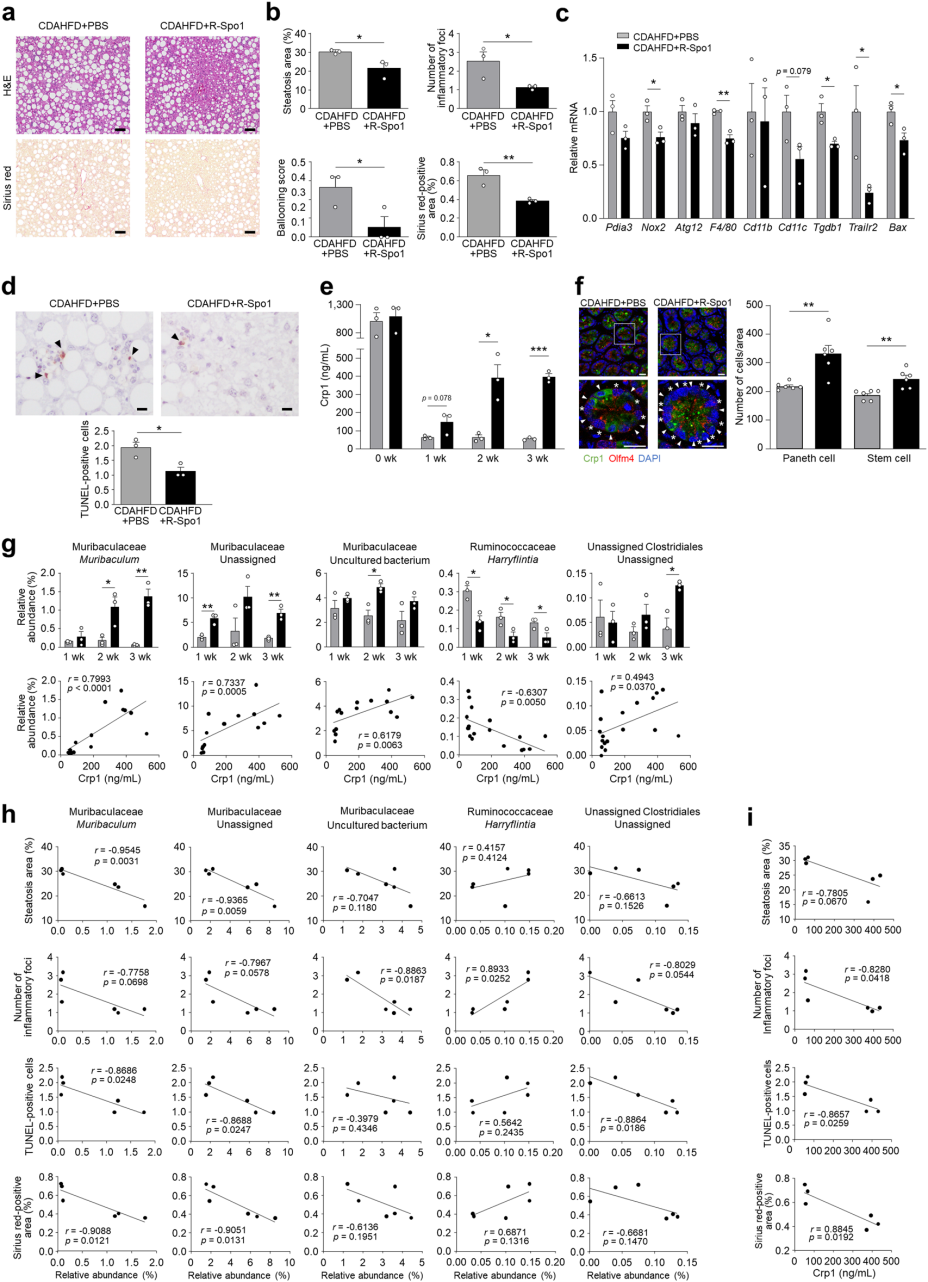
R-Spo1 restores luminal α-defensin, suppresses intestinal dysbiosis and improves NASH progression. Six-week-old C57BL/6J mice were fed CDAHFD to induce NASH and intravenously injected with R-Spo1 at a dose of 600 μg or PBS three times a week for 3 wk. (**a**) Representative images of H&E- and Sirius red-stained liver sections. Scale bars: 50 μm. (**b**) Quantification of steatosis area, lobular inflammation, hepatocellular ballooning, and Sirius red-positive area. The degree of lobular inflammation was evaluated by counting the number of inflammatory foci. (**c**) Hepatic mRNA expression of ER stress, oxidative stress, autophagy, inflammation, and apoptosis marker genes. (**d**) Representative images of TUNEL staining of liver sections and quantification of TUNEL-positive cells. Scale bars: 10 μm. (**e**) Fecal Crp1 levels in CDAHFD + PBS and CDAHFD + R-Spo1 group. (**f**) Representative images of immunofluorescence staining of Crp1 (green) and Olfm4 (red) in small intestine from CDAHFD + PBS and CDAHFD + R-Spo1 mice at 3 wk (left). Arrowheads indicate Paneth cells and asterisks indicate stem cells. Scale bar: 20 μm. Quantification of the number of Paneth cells assessed by expression of Crp1 and the number of stem cells assessed by expression of Olfm4 per ileal unit area (right). Data are shown as mean ± SEM for *n* = 6 fields/group and quantified based on at least 2 fields per mouse. Each group contains 3 mice. (**g**) Relative abundance of individual genera that were significantly recovered in CDAHFD + R-Spo1 group from CDAHFD group of 28 genera shown in Fig. 4d (upper panel). Correlation analysis between fecal Crp1 levels and relative abundance of individual genera (lower panel). (**h**) Correlation analysis between relative abundance of individual genera and NASH pathology. (**i**) Correlation analysis between fecal Crp1 levels and NASH pathology. Data are shown as mean ± SEM for *n* = 3 per group in (**b**,**c**,**d**,**e**,**g**). *P < 0.05, **P < 0.01 and ***P < 0.001, by unpaired two-tailed Student’s *t* test (**b**,**c**,**d**,**e**,**f**,**g**) and Pearson correlation test (**g**,**h**,**i**).

To clarify the association between the early reduction of luminal α-defensins and dysbiosis along with NASH pathology, we tested whether restoration of luminal α-defensins prevents NASH development. Since Wnt activator R-Spo1 enhances α-defensin secretion by stimulating Paneth cell differentiation from stem cells^27^, CDAHFD-fed mice were injected intravenously with R-Spo1 for 3 wk. Histological analyses of the liver showed that the area of steatosis, the degree of lobular inflammation and hepatocellular ballooning, and the area of fibrosis were all decreased significantly in R-Spo1-treated CDAHFD mice (CDAHFD + R-Spo1 group) compared to CDAHFD-fed mice treated with PBS (CDAHFD + PBS group) (Fig. 5a,b). R-Spo1 treatment significantly reduced hepatic mRNA expression of *Nox2*, *F4/80*, *Tgfb1*, *Trailr2*, and *Bax*, tended to reduce *Cd11c* expression in the liver, and significantly decreased the number of TUNEL-positive cells (Fig. 5c,d). Fecal Crp1 levels were measured to determine whether R-Spo1 treatment restores luminal levels of secreted Crp1. Fecal Crp1 in the CDAHFD + R-Spo1 group started to increase at 1 wk, and significantly increased after 2 wk compared to the CDAHFD + PBS group (Fig. 5e). Numbers of Paneth cells in the CDAHFD + R-Spo1 group increased significantly at 3 wk along with stem cells, indicating that R-Spo1 treatment restored fecal Crp1 levels by elevating Paneth cell numbers (Fig. 5f).

Next, we assessed whether enhanced fecal Crp1 levels by R-Spo1 treatment attenuates dysbiosis. Among the families of intestinal bacteria which increased in the CDAHFD group during disease progression (Supplementary Fig. 5a), the relative abundances of Prevotellaceae and Peptostreptococcaceae were reduced by R-Spo1 treatment (Supplementary Fig. 7a). In contrast, R-Spo1 treatment reduced the proportion of the intestinal microbiota that increased during disease progression including Muribaculaceae, unassigned Clostridiales, and unassigned family of unassigned Bacteroidia (Supplementary Fig. 7a). Fecal Crp1 levels were positively correlated with the relative abundance of Muribaculacea and unassigned Clostridiales (Supplementary Fig. 7b). At the genus level, among the intestinal microbiota that correlated with both fecal Crp1 levels and NASH pathology (Fig. 4d), the proportions of *Muribaculum,* unassigned Muribaculaceae*,* Muribaculaceae uncultured bacterium, and unassigned Clostridiales were restored by R-Spo1 treatment, showing positive correlation with Crp1 levels. The outgrowth of *Harryflintia* was inhibited, showing negative correlation with Crp1 levels (Fig. 5g). Furthermore, the number of inflammatory foci was negatively correlated with Muribaculaceae uncultured bacterium, positively correlated with *Harryflintia*, and tended to be negatively correlated with *Muribaculum*, Muribaculaceae uncultured bacterium, and unassigned genus of the unassigned Clostridiales family. Both *Muribaculum* and unassigned Muribaculaceae showed negative correlation with the steatosis area, the number of TUNEL-positive cells, and the area of fibrosis. In addition, unassigned genus of the unassigned Clostridiales family was negatively correlated with the number of TUNEL-positive cells (Fig. 5h). Fecal Crp1 levels were negatively correlated with the number of inflammatory foci, the number of TUNEL-positive cells, and the fibrosis area, indicating restoration of Crp1 secretion ameliorates NASH pathology (Fig. 5i). Taken together, enhancing luminal α-defensin secretion from Paneth cells by R-Spo1 treatment reduced liver fibrosis via ameliorating inflammation and apoptosis in the liver along with preventing dysbiosis.

### Oral administration of Crp4 suppresses dysbiosis and ameliorates liver fibrosis

**Figure 6:**
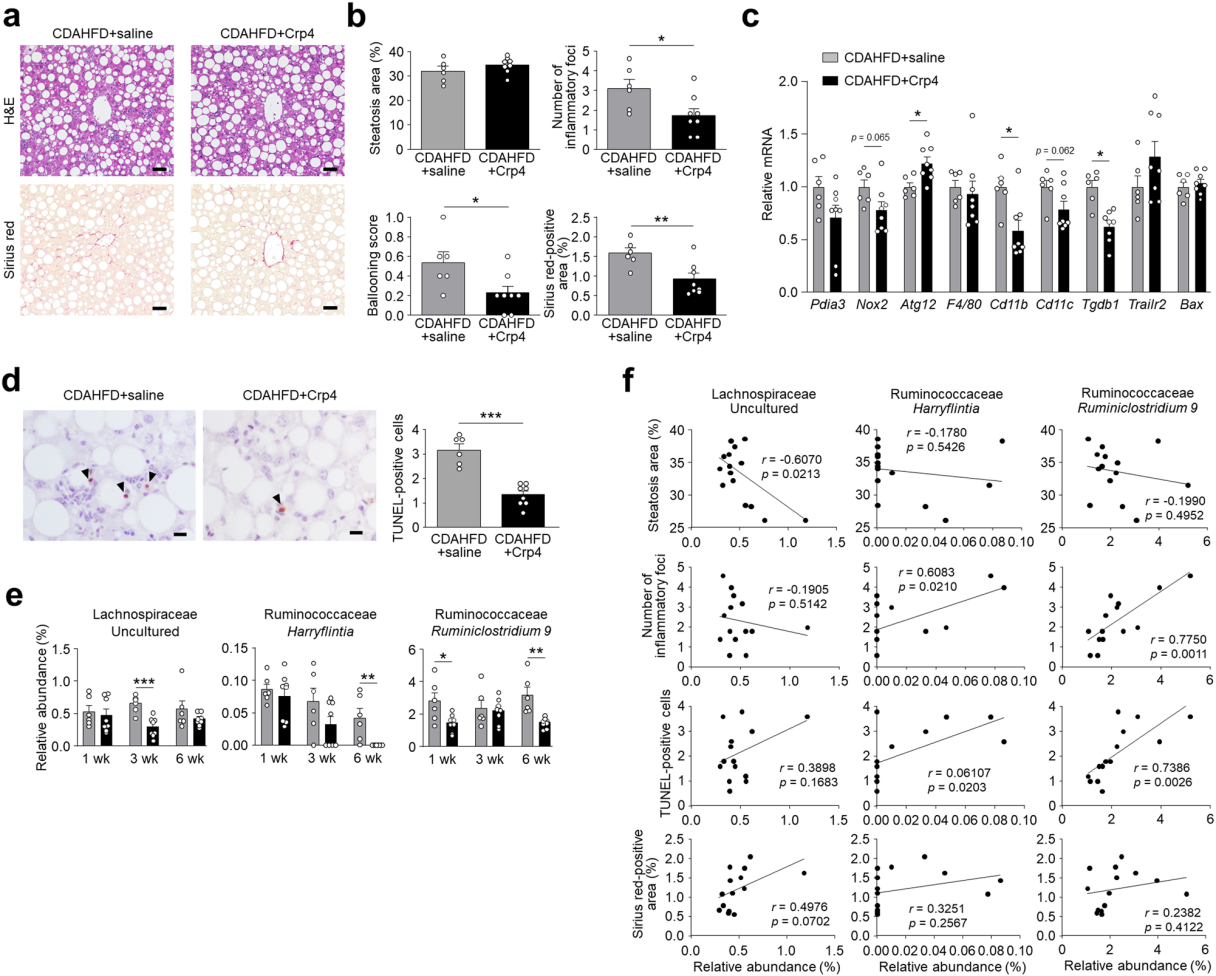
Oral administration of Crp4 increases autophagy related gene expression, suppresses dysbiosis, and ameliorates liver fibrosis. Six-week-old C57BL/6 J mice were fed CDAHFD to induce NASH and orally administered with Crp4 at a dose of 110 µg or saline twice daily for 6 wk. (**a**) Representative images of H&E- and Sirius red-stained liver sections. Scale bars: 50 µm. (**b**) Quantification of steatosis area, lobular inflammation, hepatocellular ballooning, and Sirius red-positive area. The degree of lobular inflammation was evaluated by counting the number of inflammatory foci. (**c**) Hepatic mRNA expression of ER stress, oxidative stress, autophagy, inflammation, and apoptosis marker genes. (**d**) Representative images of TUNEL staining of liver sections and quantification of TUNEL-positive cells. Scale bars: 10 µm. (**e**) Relative abundance of individual genera that were significantly recovered in CDAHFD + Crp4 group from CDAHFD group of 28 genera shown in Fig. 4d. (**f**) Correlation analysis between relative abundance of individual genera and NASH pathology. Data are shown as mean ± SEM for *n* = 6–8 per group. *P < 0.05, **P < 0.01 and ***P < 0.001, by unpaired two-tailed Student’s *t* test (**b**,**c**,**d**,**e**) and Pearson correlation test (**f**).

Finally, because granules of Paneth cells contain microbicidal components in addition to abundant α-defensins^29^, we investigated whether oral administration of an exogenous α-defensin prevents dysbiosis and subsequent NASH development. Oral administration of Crp4, which is known as the most potent in vitro bactericidal activities among Crps^31^ has been reported to improve homeostasis of the intestinal microbiota in mouse GVHD model and in chronic social defeat stress model^24,27^. Therefore, Crp4 was administered orally to CDAHFD-fed mice for 6 wk. Because C57BL/6 mouse strain lacks the Crp4 gene^32^, dosing Crp4 introduces an exogenous α-defensin, and we confirmed that orally administered Crp4 reached the intestinal lumen by measuring levels of fecal Crp4 (Supplementary Fig. 8). Histological analysis of liver revealed that oral administration of Crp4 decreased the degree of lobular inflammation and hepatocellular ballooning and the area of fibrosis significantly, though, Crp4 administration did not affect the area of hepatic steatosis (Fig. 6a,b). Analysis of liver gene expression revealed that Crp4 administration increased *Atg12* mRNA levels, decreased *Cd11b* and *Tgfb1* mRNAs, and tended to decrease levels of *Nox2* and *Cd11c* mRNAs. Genes that decreased in expression by R-Spo1 treatment including *F4/80, Trailr2*, and *Bax* were unaffected by oral Crp4 administration (Fig. 6c). TUNEL-positive cells in the liver decreased significantly in the Crp4-treated group compared to the untreated group (Fig. 6d). These findings collectively support the view that Crp4 prevented fibrosis by activating autophagy, ameliorating both inflammation and apoptosis in the liver, suggesting a different mechanism from R-Spo1 treatment.

To assess whether oral Crp4 ameliorated liver fibrosis by suppressing dysbiosis, the intestinal microbiota composition was analyzed. At the family level, among the intestinal bacteria that increased in the CDAHFD group during disease progression, the proportion of Eggerthellaceae, Streptococcaceae, and Ruminococcaceae decreased with Crp4 administration (Supplementary Fig. 9). At the genus level, the outgrowth of *Harryflintia* was suppressed by Crp4 administration comparably to R-Spo1 treatment. On the other hand, the increase of uncultured Lachnospiraceae and *Ruminoclostridium 9*, which were not reversed by R-Spo1 treatment reduced by Crp4 administration (Fig. 6e). Moreover, both *Harryflintia* and *Ruminiclostridium 9* were positively correlated with the number of inflammatory foci and TUNEL-positive cells, showing that Crp4 administration ameliorated inflammation and apoptosis accompanied by fibrosis in the liver via preventing dysbiosis (Fig. 6f). Taken together, both R-Spo1 and Crp4 prevented liver fibrosis with amelioration of liver inflammation and apoptosis, suppressing dysbiosis, though, the gene expression in the liver and the intestinal microbiota composition, which improved by Crp4 administration, were different from those by R-Spo1 treatment.

## Discussion

Fibrosis is the major determinant of mortality in patients with NASH, though, development mechanisms of the fibrosis are not fully understood yet^2^. This study focused on the “gut-liver axis” that leads to liver fibrosis and analyzed the relationship between Paneth cell α-defensins and NASH pathology via dysbiosis. The liver inflammation and apoptosis are the main factors contributing to pathology of NASH patients^4^. In this study, we confirmed that inflammation and apoptosis are key drivers of liver fibrosis in the CDAHFD model consistent with previous reports^33,34^. In NASH patients, ER stress occurs in hepatocytes with steatosis, and mitochondrial dysfunction accompanied by oxidative stress and suppression of autophagy induce and aggravate liver inflammation^4^. Among ER stress markers, *Pdia3*, whose expression increases in NASH patients^35^, increased at 3 wk, followed by elevation of *Perk* and *Chop*. In addition, *Nox2* increased, whereas *Prdx6* decreased, and autophagy-related genes including *Atg3*, *Atg12*, and *Lc3b* decreased in the CDAHFD group, suggesting excessive diet-induced oxidative stress and impairment of autophagy. Also, *Trailr2* and *Bax*, which are known to elevate due to toxic lipid-induced ER stress^36^, increased in the CDAHFD group. Our results suggested that inflammation and apoptosis in the liver accompanied by ER stress, oxidative stress, and impairment of autophagy contribute to liver pathology in the CDAHFD group, similar to NASH patients.

We showed that fecal Crp1, an abundant mouse α-defensin, significantly decreases before NASH onset along with disease progression in the CDAHFD group, and dysbiosis sharing some similar features with NAFLD/NASH patients occurs. At the family level, Prevotellaceae, previously reported to increase in NAFLD patients^37^, increased in the CDAHFD group. At the genus level, *Bacteroides*, known to increase and positively correlate with fibrosis score in NASH patients^38^, was elevated in the CDAHFD group. Although further studies are needed to address mechanisms on the intestinal hyperpermeability in detail, the CDAHFD group also showed elevated serum zonulin levels and bacterial translocation similar to NASH patients. The composition of the intestinal microbiota is affected by varied factors including diet, factors in intestinal environment, and host-derived factors^39^, and the cause of dysbiosis in NASH has not been well understood. α-Defensins secreted from Paneth cells have been known to regulate the symbiotic intestinal microbiota composition by the selective bactericidal activities^18,21,22,27^. We showed that Paneth cell α-defensin secretion decreased before onset of NASH in the CDAHFD group, and the amount of α-defensin secreted into the intestinal lumen was enhanced by administration of R-Spo1, ameliorating steatosis, inflammation, apoptosis, and fibrosis in the liver. Recent studies showed that Muribaculaceae, which increased by R-Spo1 treatment, is required for inner mucus layer formation of the intestine and SCFA production, known to have beneficial effects on the host^40,41^. Furthermore, human Paneth cell α-defensin HD5 does not elicit bactericidal activities against Muribaculaceae, suggesting a promotion of colonization^42^. On the other hand, *Harryflintia*, which decreased by R-Spo1 treatment, increased in the animal model of hyperlipidemia and atherosclerosis, and positively correlated with the disease phenotype^43^, suggesting that *Harryflintia* is deleterious microbiota. Enhancing luminal α-defensin secretion by R-Spo1 treatment prevented liver fibrosis possibly by the selective microbicidal activities, increasing Muribaculaceae and decreasing *Harryflintia*, which contribute to ameliorating the disease progression.

R-Spo1 enhanced luminal secretion of α-defensin by regeneration of Paneth cells, though, it has been known that Paneth cells secrete other antimicrobial proteins including lysozyme and secretory phospholipase A_2_^14^. In addition, R-Spo1 is reported to increase not only numbers of Paneth cells but also goblet cells^27^. Goblet cells secrete mucus, which harbors the intestinal microbiota and influences its composition^44^. Therefore, we further tested whether increasing luminal α-defensin prevents NASH by oral administration of α-defensin. We used Crp4 but not Crp1 because Crp4 has the strongest microbicidal activities among Crps^31^ and also because we can distinguish administered Crp4 from endogenous α-defensins. Oral Crp4 administration prevented NASH pathology including fibrosis, and inhibited the proportion of *Harryflintia* as did R-Spo1 treatment. In contrast, Crp4 but not R-Spo1 administration decreased *Ruminiclostridium 9* and uncultured Lachnospiraceae, suggesting possible different action for the microbiota. In addition, it has been reported that *Ruminiclostridium* *9*, which was positively correlated with the number of inflammatory foci in this study, also increases in a high-fat/high-fructose diet-induced dyslipidemia model^45^. Although further studies are needed to confirm a direct association of Crp1, these findings suggest that orally administered Crp4 prevented progression of liver fibrosis by ameliorating inflammation along with inhibiting bacterial outgrowth of *Harryflintia* and *Ruminiclostridium* *9*.

The composition and function of the intestinal microbiota differ between the mucus side and the luminal side in the intestine, forming a unique ecosystem^46^. Although both R-Spo1 and Crp4 corrected dysbiosis by increasing the amount of α-defensin in the intestinal lumen, they affected the intestinal microbiota differently except for *Harryflintia*. Muribaculaceae affected by R-Spo1 is considered as the mucus resident bacteria^47^, while *Ruminiclostridium* *9*, which was affected by Crp4 is known as the luminal bacteria^48^. These findings provide an insight that R-Spo1 and Crp4 modified bacteria localized in mucus and lumen, respectively. It is possible that intravenously administered R-Spo1 enhanced α-defensin secretion from de novo Paneth cells into mucus layer and predominantly contributed to modifying mucosa-associated bacteria, whereas oral administration of Crp4 mainly affected luminal resident bacteria of the intestine. Furthermore, R-Spo1 decreased apoptosis associated genes, *Trailr2* and *Bax*, whereas Crp4 increased expression of *Atg12*, an autophagy related protein. Free fatty acids in hepatocytes have been reported to induce TRAILR2 expression by CHOP induction and apoptosis^36^. R-Spo1 prevented lipid accumulation in liver, suggesting that R-Spo1 suppresses liver fibrosis by inhibiting apoptosis induced by lipotoxicity. On the other hand, oral Crp4 administration activated autophagy with less effects on liver steatosis and gene expression of *Trailr2* and *Bax*. *Atg12* whose gene expression increased by Crp4 has a crucial role for autophagy^49^, and loss of autophagy in hepatocytes causes apoptosis, inflammation, and fibrosis in the liver^50^. These results suggest that Crp4 prevented liver fibrosis by a different mechanism of action from R-Spo1, which may relate to the differences in the improving intestinal microbiota composition.

Paneth cells regulate the composition of the intestinal microbiota by secreting granules rich in α-defensins in response to cholinergic agents, bacteria, and dietary factors^15,17,51^. Secretory responses of Paneth cells need a biphasic increase in cytosolic Ca^2+^ concentration, and Ca^2+^-activated potassium channel KCNN4 is the essential modulator of Ca^2+^ concentration during Paneth cell secretion^52^. In addition, mice having defective cystic fibrosis transmembrane conductance regulator (CFTR) show unusual accumulation of Paneth cell granules in the intestinal crypt lumen, suggesting that CFTR is important for dissolution of secreted granules^53^. Our RNA-seq analysis of Paneth cells revealed that the expression of both *KCNN4* and *CFTR* decreased in Paneth cells of the CDAHFD group. RAS-related GTP-binding protein (Rab) and soluble NSF attachment protein receptor (SNARE) family proteins, which allow vesicle transport and fusion, have key roles in granule secretion. The expression of *Rab26*, which has been reported to localize on secretory granules and required to amylase release in parotid acinar cells^54^ and vesicle-associated membrane protein 2 (VAMP2), essential for glucagon-like peptide 1 secretion in intestinal L cell^55^ decreased in the CDAHFD group. On the other hand, overexpression of syntaxin-binding protein 1 (Stxbp1) inhibits the SNARE complex assembly and decreases insulin secretion^56^. Paneth cell in the CDAHFD group showed decreased expression of *Rab26* and *Vamp2*, which are essential for granule secretion, and increased expression of *Stxbp1*, which is a negative regulator of secretory response. Furthermore, decreased granule secretion from Paneth cells in the CDAHFD group was directly revealed by visualization and quantification of granule secretory response in enteroids. Because mutation of autophagy related genes such as *Atg16L1* leads to the abnormal morphology of Paneth cell granules, autophagy elicits important roles in granule formation and secretion of Paneth cells^29^. *Optn*, which decreased in the CDAHFD group Paneth cells, has been reported to promote autophagosome formation via recruitment of Atg12-5-16L1 complex^57^. Our findings that Paneth cells in the CDAHFD group showed reduction of *Optn* expression suggests that abrogation of autophagy leads to abnormal granule formation and decreased granule secretion. Thus, it is possible that decreased granule secretion in the CDAHFD group occurs through impairment of influx of ions such as Ca^2+^, vesicle transport, and autophagy process in Paneth cells.

The relationship between diet and Paneth cell function has been reported. The excess nutrients decreased secretion of α-defensin from Paneth cells and led to dysbiosis^58^. Similarly, abnormal granule morphology and decreased expression of HD5 were reported in Paneth cells of obese individuals^59^. Furthermore, leucine and butyric acid induce secretion of α-defensin from Paneth cells^51^. These studies suggested that certain dietary factors and intestinal microbiota metabolites are directly involved in the induction of α-defensin secretion. Although CDAHFD model mice have limitations such as not showing obesity and insulin resistance usually observed in patients with NASH^34^ so that further studies are needed to clarify the roles of α-defensin in NASH, our findings are the initial discoveries providing novel insights into the process of NASH fibrosis in the “gut-liver axis” and may further suggest a novel therapeutic approach for NASH targeting Paneth cell α-defensin via regulation of the intestinal microbiota.

## Materials and methods

### Mice

Three-week-old male C57BL/6J mice were purchased from CLEA Japan Inc. and acclimated for 3 weeks prior to be using in experiments. After acclimation, six-week-old mice were divided into two groups: SD group fed with standard diet (SD; A06071314, Research Diets Inc.) and CDAHFD group fed with choline-deficient, L-amino acid-defined, high-fat diet with 0.1% methionine (CDAHFD; A06071302, Research Diets Inc.). The mice were housed on 12-h light/dark cycle and had free access to food and tap water. All animal experiments in this study were conducted after obtaining approval from the Institutional Animal Care and Use Committee of the National University Corporation at Hokkaido University in accordance with Hokkaido University Regulations of Animal Experimentation. This study was also carried out in compliance with the ARRIVE guidelines.

### Histological analysis

The left lobe of liver was rapidly excised and fixed in 10% neutral buffered formalin. 4 μm sections of paraffin-embedded tissue were stained with H&E or Sirius red. The average number of inflammatory foci and hepatocellular ballooning were obtained from 5 randomly selected fields (228 × 303 μm^2^) per slide by using image analysis software, NIS-Elements D ver. 4.13 (Nikon). Hepatocellular ballooning was scored as follow: 0 (ballooned cells are absent), 1 (ballooned cells are present). The average percentage of hepatic steatosis area and fibrosis area were assessed by quantification of lipid accumulation area and Sirius red-positive area, respectively, in at least 20 fields (272 × 361 μm^2^) per slide using the BZ-II analyzer (KEYENCE). TUNEL assay was performed using the apoptosis in situ detection kit (Wako) following manufacturer’s instruction. TUNEL-positive cells were quantified by counting positive nuclei in 5 randomly selected fields (118 × 156 μm^2^) per slide by using NIS-Elements D.

### Reverse transcription and quantitative PCR

DNA-free RNA was obtained from ileal or liver tissue using the RNeasy Mini Kit (QIAGEN) with Dnase treatment. 0.5 μg total RNA was reverse transcribed using SuperScript VILO MasterMix (Life Technologies) by thermal cycled at 25 °C for 10 min, 42 °C for 60 min, and 85 °C for 5 min using Mastercycler EP (Eppendorf). Quantitative PCR was performed using Roche LightCycler 96 (Roche) with fluorescence-labeled locked nucleic acid (LNA) hydrolysis probes (Roche) from the Universal Probe Library (UPL) following the manufacturer’s protocol. Gene expression was normalized to hypoxanthine guanine phosphoribosyl transferase-1 (*Hprt1*). The primer sequences are listed in Supplementary Table 2.

### Quantification of fecal Crps

Fecal samples were dried and powdered by using a Multi-beads shocker (Yasui Kikai). 30 mg of fecal samples was vortex mixed with 300 μL PBS for 12 h at 4 °C. Fecal suspension was centrifuged at 20,400 g for 10 min at 4 °C, and levels of Crp1 or Crp4 in supernatants were measured by sandwich ELISA as previously described^23,60^.

### Whole-mount immunofluorescent staining and image analysis

Whole-mount immunofluorescent staining was performed using a modification of a previously reported method^61^. The ileal tissue was fixed in 4% paraformaldehyde (Sigma) for 2 h at room temperature. Fixed tissue was permeabilized with 0.5% Triton X-100 (Sigma) overnight at room temperature, and then blocked with 10% goat serum (Sigma) and 0.5% Triton X-100 overnight at 4 °C. For SD and CDAHFD group, antibody reaction was performed with FITC labeled mouse anti-Crp1 antibody (50 μg/mL, clone 77-R63, produced in our laboratory) and Alexa Fluor 647-labeled anti-mouse/human CD324 (E-cadherin) antibody (1:100, clone DECMA-1, BioLegend). For CDAHFD + PBS and CDAHFD + R-Spo1 group, the primary antibody reaction was performed with rabbit anti-Olfactomedin 4 (Olfm4) antibody (1:80, clone D6Y5A, Cell Signaling) for 1 day at 4 °C, and then the secondary antibody reaction was performed with Alexa Fluor 555 conjugated F(ab′)2-goat anti-rabbit IgG (dilution 1:500, Thermo Fisher Scientific) and FITC labeled mouse anti-Crp1 antibody overnight at 4 °C. After washing, nuclei were stained with DAPI (Thermo Fisher Scientific). Samples were immersed in the optical-clearing solution (RapiClear 1.52, Sunjin Lab).

For quantification of Crp1 fluorescence intensity and counting numbers of Paneth cells and stem cells, Z-stack images were obtained using a confocal microscope (A1, Nikon) equipped with CFI Apo LWD 20X WI λS (Nikon). The number of Paneth cells was quantified by counting Crp1 immunostaining positive cells on 3 fields (150 × 150 μm^2^) per tissue. The number of stem cells was quantified counting Olfm4 positive cells on 3 fields (150 × 150 μm^2^) per tissue. Crp1 fluorescence intensity per Paneth cell was measured by creating a region of interest using image analysis software, NIS-Elements AR ver. 5.11 (Nikon), on 3 fields (150 × 150 μm^2^) per tissue, and the mean intensity per field was calculated. For quantification of the number and diameter of Paneth cell granules, Z-stack images were obtained using A1 with CFI Apo TIRF 60X Oil (Nikon). The number and diameter of Paneth cell granules were measured on 3 fields (33 × 33 μm^2^, 2 Paneth cells/field) per tissue.

### TEM

5-mm-long segments of terminal ileum were immediately fixed in 2% paraformaldehyde and 2% glutaraldehyde at 4 °C for overnight. Next, samples were post-fixed with 2% osmium tetroxide at 4 °C for 2 h. Dehydration was carried out, followed by embedding in Quetol-812 epoxy resin (Nisshin EM). After staining with 2% uranyl acetate and lead stain solution (Sigma), the ultrathin sections were examined with a JEM-1400Plus transmission electron microscope (JEOL Ltd.) at an acceleration voltage of 100 kV.

### Paneth cell RNA-seq analysis

Crypts were isolated from the small intestine of mice fed with SD or CDAHFD for 3 wk as previously described^17^. Briefly, the ileum segments were shaken in cold HBSS containing 30 mM EDTA. After vigorous shaking for ~ 300 times in HBSS, the isolated crypts were resuspended in HBSS containing 300 U/mL collagenase (Sigma), 10 µM Y-27632 (Sigma), and 1 mM *N*-acetylcysteine (Sigma), and shaken at 180 rpm for 5 min at 37 °C on a horizontal shaker (TAITEC). Then, 50 μg/μL Dnase I (Roche) was added, and the sample was mixed by pipetting. Cells were pelleted at 500 g for 5 min at 4 °C and resuspended in washing buffer (DMEM/F12 containing 10 μM Y-27632 and 1 mM *N*-acetylcysteine), then passed through 40-μm cell strainer (BD Falcon). Paneth cells were stained with Zinpyr-1 (Santa Cruz) and Allophycocyanin (APC) anti-CD24 (clone M1/69,)Abcam) in washing buffer for 10 min at 37 °C. After Paneth cell labeling, the cells were sorted by flow cytometry using a cell sorter (JSAN, Bay Bioscience). Single cells were gated by forward scatter and side scatter. Cells were sorted directly into lysis buffer for RNA isolation (PureLink RNA Mini Kit, Invitrogen). To make a pooled sample, at least 10,000 Paneth cells were sorted from 2 separate mice, and sorting experiment was repeated three times. 2 pools (each pool contains Paneth cells from 6 separate mice) were prepared for each group. Total RNA was isolated using Invitrogen® PureLink RNA Purification System according to manufacturer’s instructions, and cDNA was synthesized using a SMART-Seq. Sequencing libraries were built with the TruSeq RNA Library Prep Kit (Illumina) and then submitted to Illumina NovaSeq 6000 for 100-bp PE reads sequencing. Fragments per kilobase of transcript per million mapped reads (FPKM) values were used, genes with FPKM values below 1 were not included in the analysis, and fold change ≥ 1.5 or ≤ 0.67 was considered differentially expressed.

### Visualization and quantification of Paneth cell granule secretion

Paneth cell granule secretion was evaluated using a modification of a previously reported method^17^. Isolated crypts from the distal 20 cm of small intestine of mice fed with SD and CDAHFD for 3 wk were embedded in Matrigel (Corning) on a collagen-coated 8 well chamber cover (Matsunami). After Matrigel polymerization, the enteroid culture media were added and incubated for 1 h at 37 °C, 5% CO_2_. The enteroids were stimulated by 1 μM of CCh (Sigma). The differential interference contrast images of Paneth cells before and 10 min after adding CCh were obtained from confocal microscopy (A1, Nikon). To quantify Paneth cell granule secretion, area of the granules at pre-and post-stimulation was measured by using the image analysis software, NIS-Elements AR, and calculated percent granule secretion. Paneth cells in 10 crypts of each mouse (4 mice of each group) were evaluated.

### Fecal DNA extraction

Fresh fecal samples were collected immediately after excretion, snap-frozen on dry ice, and stored at − 80 °C. For total DNA extraction, 200 mg fecal samples were processed by QIAamp Fast DNA Stool Mini Kit (QIAGEN) according to the manufacturer’s protocol. Final DNA concentrations were quantified from the absorption at 260 nm with a Nanodrop 2000 spectrometer (Thermo Fisher Scientific).

### 16S rDNA sequencing

The V3-V4 variable region of 16S ribosomal RNA genes were amplified from fecal DNA extracts using universal primer set of Bakt 341F (5-cctacgggnggcwgcag) and Bakt 805R (5-gactachvgggtatctaatcc)^62^. PCR amplification was performed using a 25 μL reaction volume mixtures comprising 0.5 ng/μL of DNA template, 200 nM of each primer and 1 × KAPA HiFi Hot Start Ready Mix (Kapa Biosystems) under the following conditions: initial denaturation at 95 °C for 3 min, 25 cycles of 95 °C for 30 s, 55 °C for 30 s and 72 °C for 30 s, followed by final extension at 72 °C for 5 min. Amplification products were subsequently purified using AMPure XP beads (Beckman Coulter). Then, index PCR was performed using a 50 μL reaction volume mixtures comprising 5 μL of purified PCR products, 5 μL of each index primer containing adapter sequence and sample specific 8 bp barcodes in the Nextera XT Index Kit v2 Set B (Illumina) and 1 × KAPA HiFi Hot Start Ready Mix under the following conditions: 95 °C for 3 min, 8 cycles of 95 °C for 30 s, 55 °C for 30 s and 72 °C for 30 s, followed by 72 °C for 5 min. Resulting amplification products were purified using AMPure XP beads, then quantified using the Qubit dsDNA HS Assay Kit (Invitrogen), and finally adjusted to 4 nM. Each amplicon was pooled and subjected to quantitative PCR using KAPA Library Quantification Kit Lightcycler 480 qPCR Mix (Kapa Biosystems), denatured following Illumina’s guideline, and then adjusted to 4 pM. Amplicon library was combined with 5% of 4 pM PhiX Control v3 (Illumina) and subjected to pair-end sequencing using MiSeq instrument with a MiSeq 600-cycle v3 kit (Illumina). Resulting sequence reads were filtered for read quality, basecalled, and demultiplexed using bcl2fastq software (Illumina).

### 16S rDNA-based taxonomic analysis

Taxonomic analysis of FASTQ files generated from MiSeq was conducted by QIIME2 software (version 2019.7)^63^. Quality-filtering, denoising, and removal of chimeric sequences were carried out by DADA2 plugin^64^ using following parameters; –p-trim-left-f 17, –p-trim-left-r 21, –p-trunc-len-f 280, –p-trunc-len-r 200, –p-max-ee-f 2 –p-max-ee-r 2. Phylogenetic tree was constructed with FastTree^65^ after alignment with MAFFT^66^. Each feature was taxonomically assigned by a naïve-bayes classifier based on 99% sequence identity to the SILVA database (v.132). α-Diversity (observed OTUs, PD whole tree, Shannon index and Simpson index) and β-diversity (weighted UniFrac distance) were calculated by Qiime2 pipeline. Statistical significance of β-diversity was determined by PERMANOVA test in Qiime2 pipeline.

### Measurement of serum zonulin

Zonulin concentration in serum was quantified by Mouse Haptoglobin ELISA Kit (Abcam, ab157714). The assay was performed according to the manufacture’s recommended methods.

### Bacterial translocation

To quantitate and identify bacteria, the spleen was removed and immediately placed into 500 μL of sterile Luria–Bertani (LB) medium. The spleen then was homogenized with a BioMasher (Nippi), and 200 μL was plated on LB agar plates and cultured either under aerobic conditions for 24 h or anaerobic conditions for 48 h at 37 °C. Colony-forming unit (CFU) were counted and calculated per organ.

### Administration of R-Spo1 and Crp4

Recombinant human R-Spo1 provided from Kyowa Kirin Co., Ltd was generated as previously reported^25,26^. R-Spo1 was intravenously administered at a dose of 600 μg three times a week for 3 wk. Recombinant Crp4, produced and purified as previously described^67^, was orally administered at a dose of 110 μg twice daily for 6 wk.

### Statistics

Data were analyzed using GraphPad Prism 8 software (GraphPad software), and results were expressed as individual points with mean values and error bars representing SEM. Statistical significance between 2 groups was determined by unpaired two-tailed Student’s *t* test. Pearson’s test was used to analyze the correlation. A *P* value of less than 0.05 was considered statistically significant.

## Supplementary Information

Supplementary Information.

## Data Availability

Paneth cell RNA-seq data were deposited in the NCBI’s Sequence Read Archive (SRA PRJNA747067). 16S rRNA sequences were uploaded to the NCBI’s SRA (PRJNA747068).
