# Supplementary material for: Decreased Paneth cell α-defensins promote fibrosis in a choline-deficient L-amino acid-defined high-fat diet-induced mouse model of nonalcoholic steatohepatitis via disrupting intestinal microbiota
Source: Sci Rep. 2023 Mar 9;13:3953. doi: 10.1038/s41598-023-30997-y (PMC9998432; doi:10.1038/s41598-023-30997-y)
Supplement: Supplementary file 1 — Supplementary Information. [file 41598_2023_30997_MOESM1_ESM.pdf]

Supplementary Information

Decreased Paneth cell α-defensins promote fibrosis in a choline-deficient L-amino acid-defined high-fat diet-induced mouse model of nonalcoholic steatohepatitis via disrupting intestinal microbiota

Shunta Nakamura [1,2], Kiminori Nakamura[1,3*], Yuki Yokoi[1,3], Yu Shimizu[3], Shuya Ohira[1], Mizu Hagiwara[1], Zihao Song[1], Li Gan[1], Tomoyasu Aizawa[1,4], Daigo Hashimoto[5], Takanori Teshima[5], Andre J. Ouellette[6], and Tokiyoshi Ayabe[1,3*]

[1]Graduate School of Life Science, Hokkaido University, Sapporo, Japan. [2]Hokkaido Laboratory, Corporate Research & Development Division, Nitto Denko Corporation, Sapporo, Japan. [3]Department of Cell Biological Science, Faculty of Advanced Life Science, Hokkaido University, Sapporo, Japan. [4]Department of Advanced Transdisciplinary Science, Faculty of Advanced Life Science, Hokkaido University, Sapporo, Japan. [5]Department of Hematology, Faculty of Medicine, Hokkaido University, Sapporo, Japan. [6]Department of Pathology and Laboratory Medicine, Keck School of Medicine, University of Southern California, Los Angeles, California, USA.

Supplementary Figures

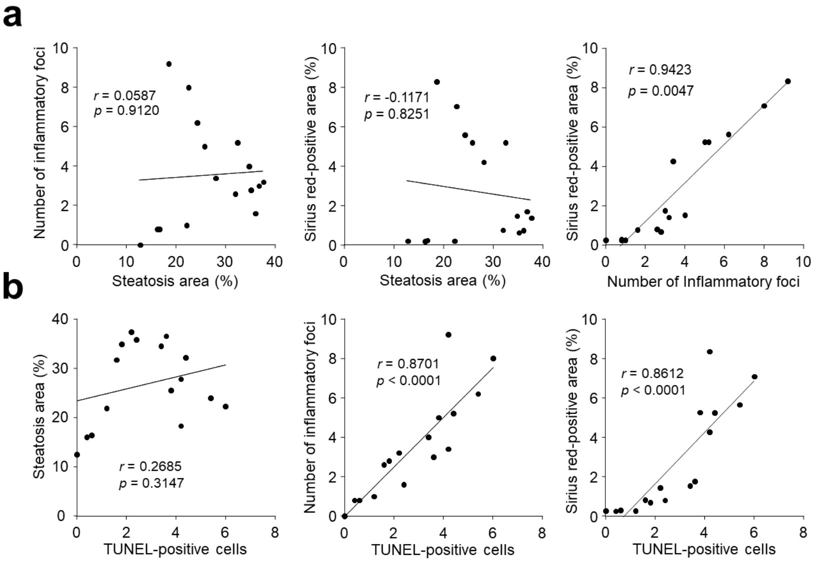

Supplementary Figure 1. Progression of liver fibrosis in the CDAHFD group is associated with hepatic inflammation and apoptosis. a Correlation analysis between steatosis area, the number of inflammatory foci and fibrosis area using CDAHFD group data at 1, 3, 6, and 12 wk. **b** Correlation analysis between the number of TUNEL-positive cells, steatosis area, the number of inflammatory foci and fibrosis area using CDAHFD group data at 1, 3, 6, and 12 wk. (**a** and **b**) *r* and *P* values by Pearson correlation test are presented.

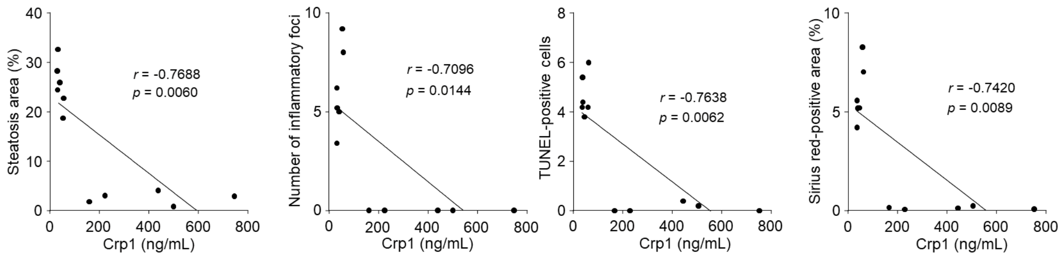

Supplementary Figure 2. Decreased secretion of Crp1 in intestinal lumen is associated with hepatic steatosis, inflammation, apoptosis, and fibrosis. Correlation analysis between fecal Crp1 levels and NASH pathology including steatosis area, the number of inflammatory foci, the number of TUNEL positive cells, and Sirius red-positive area using SD and CDAHFD group data at 12 wk. *r* and *P* values by Pearson correlation test are presented.

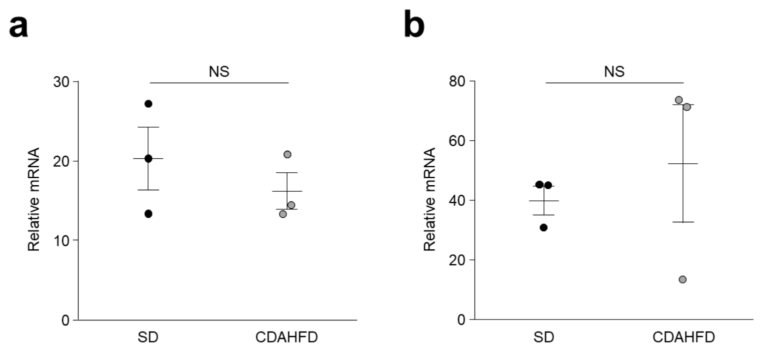

Supplementary Figure 3. Expression of Crp1 is not affected by CDAHFD. mRNA levels of Crp1 in ileum of SD and CDAHFD group at 3 wk (**a**), and 6 wk (**b**) analyzed by real time-PCR. Data are shown as mean ± SEM for $n = 3$ per group. The data represent mean ± SEM. *$P < 0.05$, by unpaired 2-tailed Student's t test. NS, not significant.

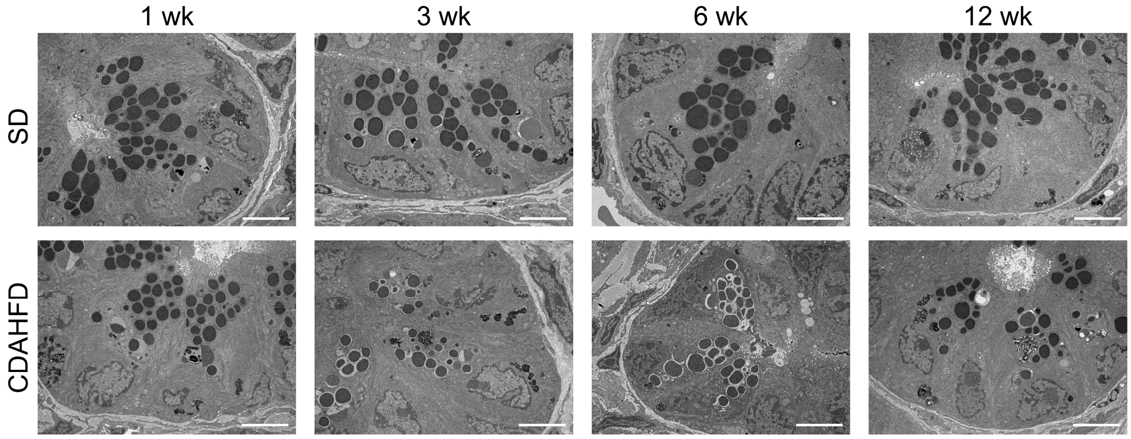

Supplementary Figure 4. Abnormal Paneth cell granules are observed in the CDAHFD group. Representative images of transmission electron microscopy of Paneth cells at the base of the ileal crypts in SD and CDAHFD group at 1, 3, 6, and 12 wk. Paneth cells in the SD group have tightly packed electron-dense core granules, in contrast, Paneth cells in the CDAHFD group have fewer loosely packed granules with a halo appearance. *n* = 1 for each group. Scale bars: 5 µm.

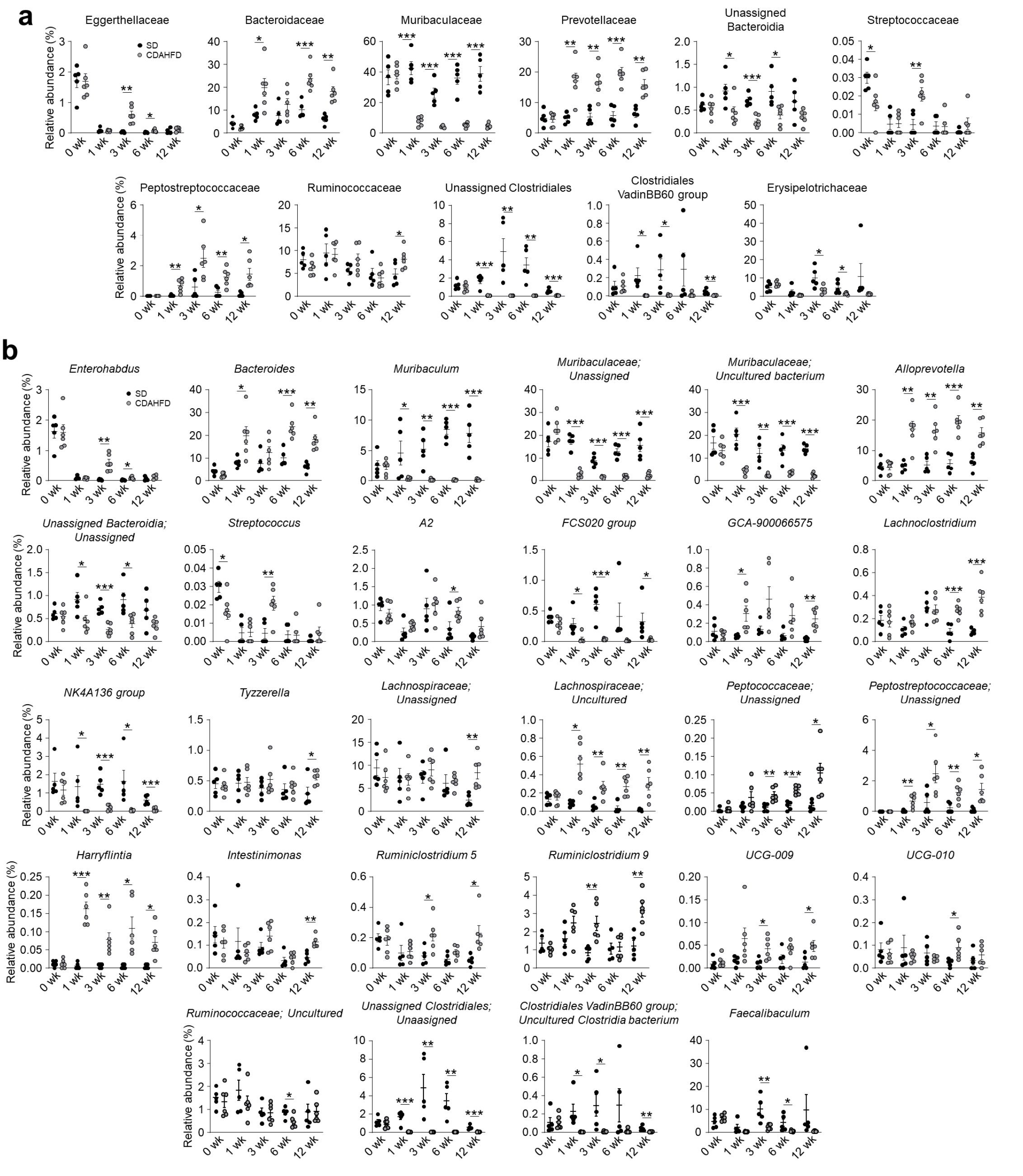

Supplementary Figure 5. Phylogenetic comparison of the intestinal microbiota in SD and CDAHFD groups. Relative abundance of individual genera that were significantly different between the SD and CDAHFD groups at the family (**a**) and genus level (**b**). Data are shown as mean ± SEM for n = 5–6 per group. *P < 0.05, **P < 0.01 and ***P < 0.001, by unpaired 2-tailed Student's t test.

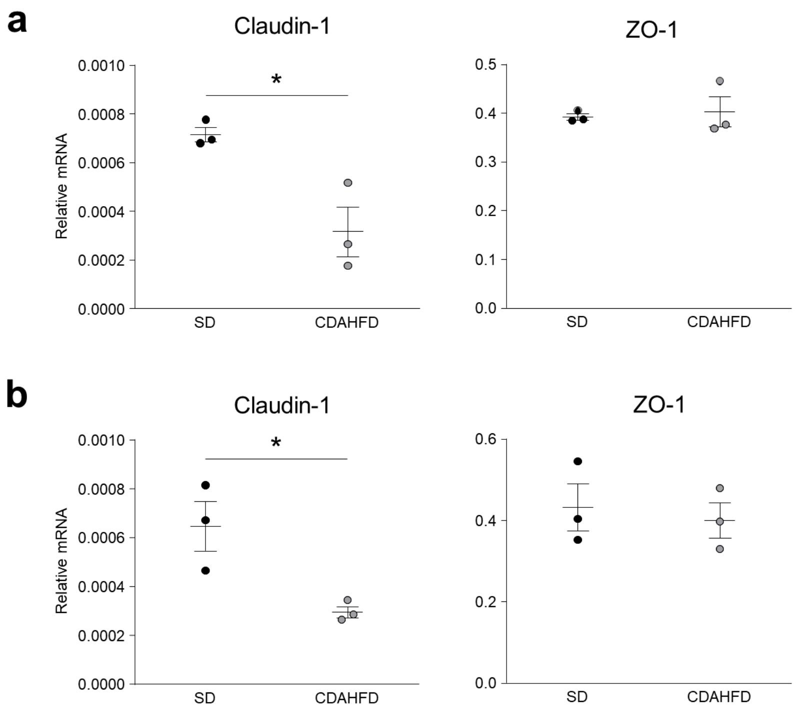

Supplementary Figure 6. mRNA expression of clauidn-1 decreased in the ileum of the CDAHFD group. mRNA expression of tight junction proteins, claudin-1 and ZO-1, in the ileum of SD and CDAHFD group at 3 wk (**a**) and 6 wk (**b**) analyzed by real-time PCR. *$P < 0.05$, **$P < 0.01$ and ***$P < 0.001$, by unpaired 2-tailed Student's t test.

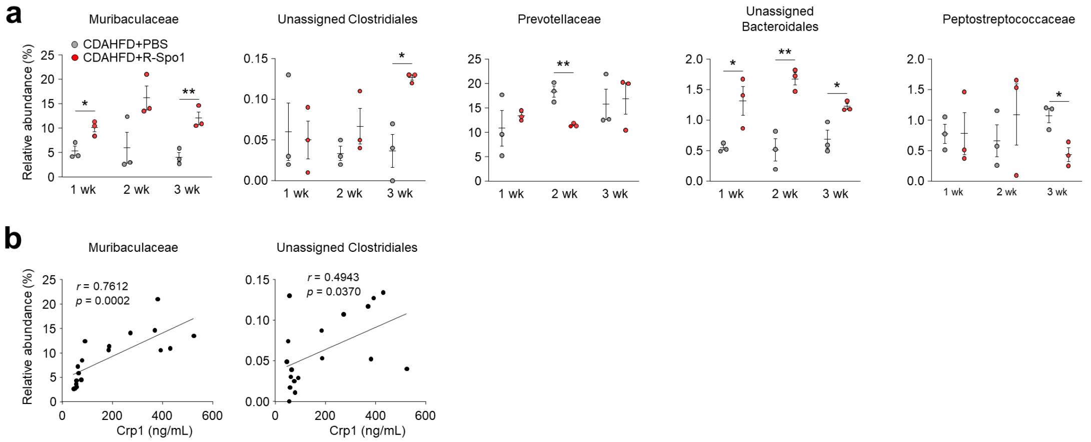

Supplementary Figure 7. R-Spo1 treatment prevents intestinal dysbiosis at the family level. a Relative abundance of individual genera that were significantly recovered in CDAHFD+R-Spo1 group from CDAHFD mice of 11 family in supplementary Fig. 5a. Data are shown as mean ± SEM for $n = 3$ per group. *$P < 0.05$, **$P < 0.01$ and ***$P < 0.001$, by unpaired 2-tailed Student's t test. **b** Correlation analysis between fecal Crp1 levels and relative abundance of individual genera CDAHFD+PBS and CDAHFD+R-Spo1 group. r and P values by Pearson correlation test are presented.

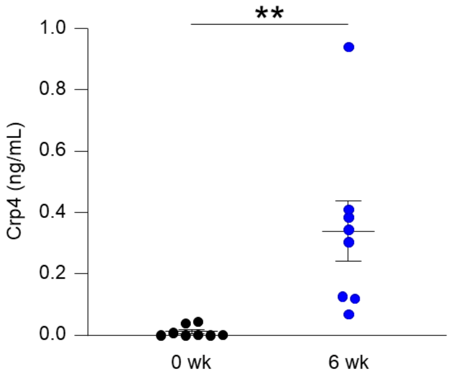

Supplementary Figure 8. Oral administered Crp4 is accumulates in the intestinal lumen. Six-week-old C57BL/6J mice were fed with CDAHFD to induce NASH and orally administered with Crp4 at a dose of 110 μg twice daily for 6 weeks. Fecal levels of Crp4 in CDAHFD+Crp4 group were analyzed by sandwich ELISA at 0 and 6 wk. Data are shown as mean ± SEM for $n = 8$ per group. *$P < 0.05$, **$P < 0.01$ and ***$P < 0.001$, by unpaired 2-tailed Student's t test.

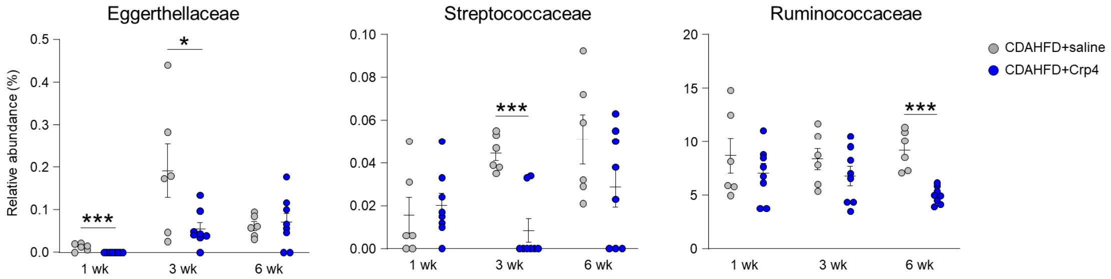

Supplementary Figure 9. Oral administration of Crp4 attenuates intestinal dysbiosis at family level. Relative abundance of individual genera that were significantly recovered in CDAHFD+Crp4 group from CDAHFD group of 11 family in Supplemental Fig. 5a. Data are shown as mean ± SEM for n = 6–8 per group. *P < 0.05, **P < 0.01 and ***P < 0.001, by unpaired 2-tailed Student's t test.

Supplementary Table 1

Differentially expressed genes and their functional clustering associated with Paneth cell function measured by RNA-seq in Paneth cells of the SD and CDAHFD group.

Function	Entrez Gene ID	Gene symbol	Description	Log_2 (CDAHFD/SD)
Ion transporter	12351	*Car4*	carbonic anhydrase 4	1.4896
	381812	*Cracr2a*	calcium release activated channel regulator 2A	-0.5982
	12638	*Cftr*	cystic fibrosis transmembrane conductance regulator	-0.8138
	16535	*Kcnq1*	potassium voltage-gated channel, subfamily Q, member 1	-0.8197
	16534	*Kcnn4*	potassium intermediate/small conductance calcium-activated channel, subfamily N, member 4	-1.0385
Oxidative stress	69065	*Chac1*	ChaC, cation transport regulator 1	0.8618
	11865	*Arntl1*	aryl hydrocarbon receptor nuclear translocator-like 1	-2.0807
Glycosylation	21982	*Tmem165*	transmembrane protein 165	0.6842
Secretion	20910	*Stxbp1*	syntaxin binding protein 1	0.6202
Vesicle transporter	71648	*Optn*	optineurin	-0.6083
	76510	*Trappc9*	trafficking protein particle complex 9	-0.6181
	22318	*Vamp2*	vesicle-associated membrane protein 2	-0.6436
	320615	*Dop1a*	DOP1 leucine zipper like protein A	-0.6872
	328778	*Rab26*	RAB26, member RAS oncogene family	-0.7285
	80877	*Lrba*	LPS-responsive beige-like anchor	-0.7782
	76688	*Arfrp1*	ADP-ribosylation factor related protein 1	-0.7875
	260302	*Gga3*	golgi associated, gamma adaptin ear containing, ARF binding protein 3	-0.8248
Autophagy	226541	*Klhl20*	kelch-like 20	-0.6205
	101613	*Nlrp6*	NLR family, pyrin domain containing 6	-0.6714
	11781	*Ap4m1*	adaptor-related protein complex AP-4, mu 1	-0.7185
	12217	*Bsn*	bassoon	-0.7440

Supplementary Table 2

PCR primers

Gene	Primer sequence (5'-3')	Universal Probe Library
Pdia3	F: TGGCCACTGTAAGAATCTGGA<br>R: TGACAATATTTGGATCTTTGCTG	#33
Perk	F: CCTTGGTTTCATCTAGCCTCA<br>R: ATCCAGGGAGGGGATGAT	#106
Chop	F: GCGACAGAGCCAGAATAACA<br>R: GATGCACTTCCTTCTGGAACA	#91
Nox2	F: GATGCACTTCCTTCTGGAACA<br>R: GTGCACAGCAAAGTGATTGG	#20
Prdx6	F: TTTCAATAGACAGTGTTGAGGATCA<br>R: CCGTGGGTGTTTCACCAT	#1
Atg3	F: GTACCTGACCCCGGTCCT<br>R: TTGGACAGTGGTGGACTAAGTG	#78
Atg12	F: CATTGACTTCATCAAAAAGTTCCTT<br>R: GGCAAAGGACTGATTCACATAA	#49
Lc3b	F: CCCCACCAAGATCCCAGT<br>R: CGCTCATGTTCACGTGGT	#7
F4/80	F: GGAGGACTTCTCCAAGCCTATT<br>R: AGGCCTCTCAGACTTCTGCTT	#42
Cd11b	F: CAATAGCCAGCCTCAGTGC<br>R: GAGCCCAGGGGAGAAGTG	#76
Cd11c	F: AGCCTCAAGACAGGACATCG<br>R: TGAATCCTGGAGGGGATCT	#106
Tgfb1	F: CTGGGCACCATCCATGAC<br>R: CAGTTCTTCTCTGTGGAGCTGA	#68
Trailr2	F: CCCTGAGATCTGCCAGTCAT<br>R: TTTCTCTGGGGGTACAGGAA	#103
Bax	F: GAACCATCATGGGCTGGA<br>R: GGTCCCGAAGTAGGAGAGGA	#69
Crp1	F: CCAAAACACAGATGAAGAGACTAAAA<br>R: GCATACCAGATCTCTCAACGATT	#68
Claudin-1	F: ACTCCTTGCTGAATCTGAACAGT<br>R: GGACACAAAGATTGCGATCAG	#18
ZO-1	F: TGCAGACCCAGCAAAGGT<br>R: GGTTTTGTCTCATCATTTCTTCAG	#12
HPRT-1	F: TCCTCCTCAGACCGCTTTT<br>R: CCTGGTTCATCATCATCGGCTAATC	#95
